# Role of metastasis-induced protein S100A4 in human non-tumor pathophysiologies

**DOI:** 10.1186/s13578-017-0191-1

**Published:** 2017-11-25

**Authors:** Fei Fei, Jie Qu, Chunyuan Li, Xinlu Wang, Yuwei Li, Shiwu Zhang

**Affiliations:** 1 Nankai University School of Medicine, Nankai University, Tianjin, 300071 People’s Republic of China; 2 Departments of Pathology, Tianjin Union Medical Center, Tianjin, 300121 People’s Republic of China; 3 Graduate School, Tianjin University of Traditional Chinese Medicine, Tianjin, 300193 People’s Republic of China; 4 Departments of Colorectal Surgery, Tianjin Union Medical Center, Tianjin, 300121 People’s Republic of China

**Keywords:** S100A4, Inflammation, Fibrosis, Epithelial–mesenchymal transition

## Abstract

S100A4, an important member of the S100 family of proteins, is best known for its significant role in promoting cancer progression and metastasis. In addition to its expression in tumors, upregulation of S100A4 expression has been associated with various non-tumor pathophysiology processes. However, the mechanisms underlying the role of S100A4 remain unclear. Activated “host” cells (fibroblasts, immunocytes, vascular cells, among others) secrete S100A4 into the extracellular space in various non-tumor human disorders, where it executes its biological functions by interacting with intracellular target proteins. However, the exact molecular mechanisms underlying these interactions in different non-tumor pathophysiologies vary, and S100A4 is likely one of the cross-linking factors that acts as common intrinsic constituents of biological mechanisms. Numerous studies have indicated that the S100A4-mediated epithelial–mesenchymal transition plays a vital role in the occurrence and development of various non-tumor pathophysiologies. Epithelial–mesenchymal transition can be categorized into three general subtypes based on the phenotype and function of the output cells. S100A4 regulates tissue fibrosis associated with the type II epithelial–mesenchymal transition via various signaling pathways. Additionally, S100A4 stimulates fibroblasts to secrete fibronectin and collagen, thus forming the structural components of the extracellular matrix (ECM) and stimulating their deposition in tissues, contributing to the formation of a pro-inflammatory niche. Simultaneously, S100A4 enhances the motility of macrophages, neutrophils, and leukocytes and promotes the recruitment and chemotaxis of these inflammatory cells to regulate inflammation and immune functions. S100A4 also exerts a neuroprotective pro-survival effect on neurons by rescuing them from brain injury and participates in angiogenesis by interacting with other target molecules. In this review, we summarize the role of S100A4 in fibrosis, inflammation, immune response, neuroprotection, angiogenesis, and some common non-tumor diseases as well as its possible involvement in molecular pathways and potential clinical value.

## Background

In humans, the S100 protein family contains 21 members that exhibit a high degree of sequence and structural similarity. These protein members are not functionally interchangeable and thus are involved in a wide range of biological processes, such as cell division, differentiation, apoptosis, proliferation, migration, and invasion; cytoskeletal organization; and enzyme activity [1–3]. The first member of the S100 protein family was documented in 1965, and the term S100 was used to refer to the solubility of this protein with a molecular mass of approximately 10 kDa in saturated ammonium sulfate solution [4]. S100 proteins have a high degree of homology with similar amino acid sequences and 3D structures. These structures are characterized by a symmetrical homodimeric fold containing two EF-hand helix-loop-helix Ca^2+^-binding domains in each monomer [5]. The carboxy-terminal EF-hand loop is a canonical Ca^2+^-coordinating site consisting of 12 amino acid residues. In contrast, the amino-terminal pseudo-EF-hand loop comprising 14 amino acid residues is considered a unique feature of S100 proteins [6, 7]. Most S100 proteins can act as Ca^2+^ sensors based on their role in signal transduction and also contribute to fluctuations in intracellular Ca^2+^ levels to cellular responses; this sensory capability is a result of changes in the S100 protein conformation following Ca^2+^ binding [2, 8]. Despite the high sequence similarity among S100 proteins, individual family members show unique characteristics, which are determined by cell- and tissue-specific expression as well as their varying biochemical properties, including affinity for lipid and divalent metal ions, conformation after oligomerization or heterodimerization, and post-translational modifications [3, 9].

S100A4 is categorized as a metastasis-associated protein and is also known as metastasin, pEL-98, 18A2, p9Ka, CAPL, calvasculin, and fibroblast-specific protein [10–12]. The human S100A4 protein is encoded by *S100A4*, which is located within a frequently rearranged gene cluster on chromosome 1q21. The gene contains four exons encoding the two EF-hands of the Ca^2+^-binding regions of S100A4 and its 101 amino acids with a molecular mass of approximately 12 kDa [13]. Binding of Ca^2+^ ions causes a conformational change in S100A4 to form a hydrophobic pocket for the recognition and combination of target molecules [14]. The original cloning efforts reported S100A4 to be a highly expressed transcript in cells with growth-related transformation [15], metastatic tumor cells [10], and in cells undergoing conversion from an epithelial to mesenchymal phenotype [16]. In addition, S100A4 is expressed in several highly motile cell types, such as T-lymphocytes, neutrophils, and macrophages, as well as in tissues, including the spleen, thymus, and bone marrow [17]. Numerous intracellular target proteins, including non-muscle myosin IIA, p53, and liprin-β1, combine with S100A4 to form complexes that regulate cell motility [18–20]. Moreover, S100A4 and several other S100 proteins were reported to be secreted into the extracellular space by various cells involved in the process of neurite outgrowth [21], angiogenesis [22], and chemokine and cytokine-like activities [23, 24].

A growing number of studies have demonstrated the direct involvement of S100A4 in cancer progression and metastasis. The expression of S100A4 in tumor cells is strongly correlated with an aggressive metastatic phenotype [10]. In humans, upregulation of S100A4, both in tumor and stroma cells, was associated with poor prognosis and low survival of patients with cancer [25, 26]. In its role as a metastasis regulating factor, expression of S100A4 has been reported to be affected by methylation, β-catenin, and extracellular factors including epidermal growth factor, tumor necrosis factor alpha (TNF-α), etc. [27]. In addition to the role of S100A4 in cancer progression and metastasis, this protein was recently found to be involved in some vital non-tumor pathophysiologies in humans, which has attracted much attention [28, 29]. Here, we summarize the role of S100A4 in non-tumor pathophysiologies, including fibrosis, inflammation, immune responses, neuroprotection, and angiogenesis, and some common non-tumor diseases as well as the possible molecular pathways in which S100A4 is involved and its potential clinical value.

## Underlying mechanisms of S100A4 in non-tumor pathophysiologies

The protein S100A4 is closely associated with both non-tumor and tumors. In addition to being expressed in tumor cells, S100A4 is expressed in many normal cells, including fibroblasts, macrophages, lymphocytes, neutrophils, vascular cells, and bone marrow-derived cells [17, 30, 31]. Several recent publications have demonstrated an association between S100A4 and non-tumor pathophysiologies, such as tissue fibrosis, inflammation, immune reaction, neuroprotection, and cardiovascular events. However, the underlying mechanisms by which S100A4 is involved in these pathophysiologies remain unclear.

A study has indicated that the S100A4-mediated epithelial–mesenchymal transition (EMT) program plays a vital role in the occurrence and development of various diseases, including tumor and non-tumor diseases [32]. The EMT is a complex of molecular programs that occurs during embryogenesis, inflammation, tissue fibrosis, and cancer progression and metastasis. It can be categorized into three  general subtypes as described in an EMT meeting at Cold Spring Harbor Laboratory in 2008 [33]. The three types of EMT are recognized depending on the phenotype of the output cells. Type I EMT (normal tissue development) involves the transition of primordial epithelial cells into motile mesenchymal cells that eventually form the basic body of gastrulation and induce neural crest cell migration. These mesenchymal cells are then re-induced as secondary epithelial cells in mesodermal and endodermal organs after undergoing mesenchymal–epithelial transition. Type II EMT (pathological conditions) involves the transition of secondary epithelial or endothelial cells to resident or inflammation-induced fibroblasts, which occurs in response to persistent inflammation and fibrosis and is regulated by the high expression of S100A4 via various signaling pathways. Type III EMT is part of the metastatic process in which epithelial carcinoma cells in their primary nodule forms migrate to a distant site via blood circulation to reform as a secondary tumor nodule [34].

**Fig. 1:**
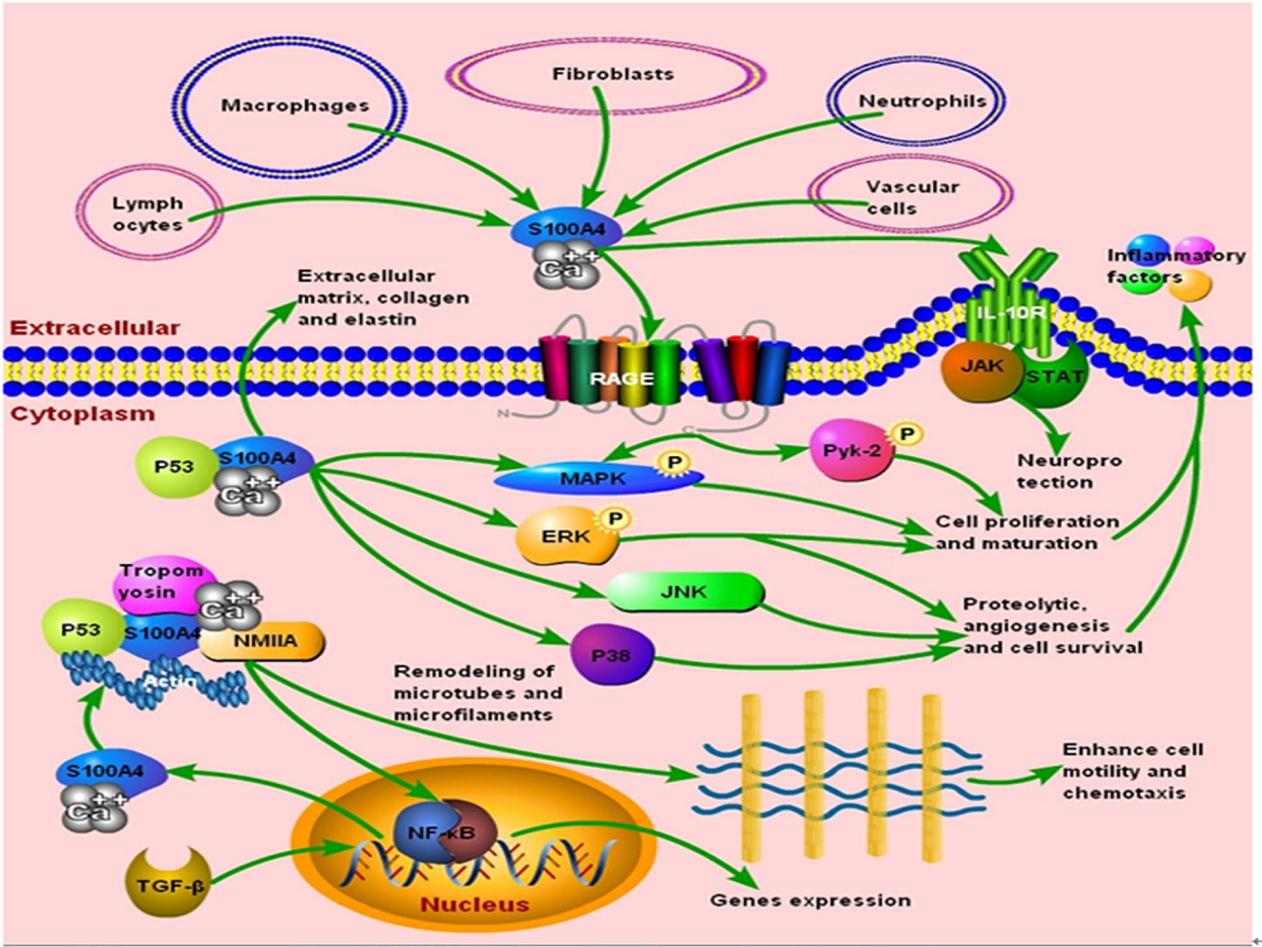
Extra-S100A4 can be released into the extracellular space by fibroblasts, macrophages, lymphocytes, neutrophils, vascular cells, and other bone marrow derived cells. The S100A4 interacted with IL-10R exerts a neuron pro-survival effect under various injuries via JAK/STAT pathway partially. Besides, the expression of extra-S100A4 leads to increasing phosphorylation of Pyk-2, MAPKs, and activating NF-κB through the RAGE-dependent regulation associated with cell migratory abilities and chemotaxis. On the other hand, the intracellular S100A4 can combine with numerous target molecules, such as NMIIA, tropomyosin, P53, and actin, to form the complexes, facilitating the remodeling of microtubes and microfilaments to enhance cell motility and chemotaxis, contributing to the infiltration of fibroblasts, immune and vascular cells into the affected region releasing inflammatory factors. In addition, S100A4 colocalizes with P53 promoting cell proliferation and collagen expression via MAPK activation and phosphorylation of ERK. The TGF-β-mediated process induces the up-regulation of S100A4 promoting the generation of extracellular matrix, collagen, elastin and others underlying the basis for the course of inflammation tissue fibrosis. Moreover, the intracellular S100A4 regulates its upstream and/or downstream gene expression involved in proteolytic activity, angiogenesis and cell survival by modulating signal pathways of MAPKs, ERK, p38, JNK, NF-κB, and p53

In addition, S100A4 enhances the motility of macrophages, neutrophils, and leukocytes and promotes the recruitment and chemotaxis of these inflammatory cells to regulate inflammation and immune [35]. S100A4 also exerts a neuroprotective pro-survival effect on neurons by rescuing them from brain injury [36] and participates in angiogenesis via its interaction with other target molecules [37]. Intracellularly, S100A4 binds to several target molecules, which leads to changes in cytoskeletal dynamics and promotion of cell motility and proliferation [38]. Moreover, various types of cells, including fibroblasts, macrophages, and lymphocytes, express and release S100A4 into their extracellular space as an active extracellular factor; thus, S100A4 has a great tendency to regulate gene expression associated with proteolytic activity, angiogenesis, cell survival, and motility by modulating the signaling pathways of mitogen-activated protein kinases (MAPKs), extracellular signal-regulated kinase, p38, Jun N-terminal kinases, nuclear factor-kappa B (NF-κB), and p53 [39–41]. The role of S100A4 in human non-tumor pathophysiologies is summarized in Fig. 1.

### S100A4 promotes tissue fibrosis

S100A4 is considered a specific fibroblast maker, and thus is frequently used to monitor or predict the mechanism of tissue fibrosis [42]. As a filament-associated protein, it is expressed in kidney fibrosis cells with fibroblast phenotypes, where it aids in conducting type II EMT [16]. All fibroblasts  observed to express S100A4 through genetic screens are likely to undergo type II EMT; thus, the protein is involved in adult epithelial or endothelial cells’ transition into fibroblasts [16]. Studies based on cell lineage-tracing have provided evidence for this transition during the formation of fibroblasts in the renal tissue [43] as well as in liver [44], lung [45], and heart [46] tissues. As in epithelial cells, endothelial cells are also sources of new fibroblasts in adult tissue, which we reported after observing that S100A4-positive fibroblasts underwent type II EMT in the heart  [46] and kidney [47]. The elevated expression of S100A4 in tubular epithelial cells contributes to their transformation to interstitial fibroblasts during kidney injury [46] and fibrosis [43]. Along with other marker proteins, such as vimentin, α-smooth muscle actin, and matrix metalloproteinases (MMPs), S100A4 is now accepted as an important hallmark of the EMT process. The expression of intracellular S100A4 is upregulated during the TGF-β-mediated transition of epithelial cells associated with the EMT program [48, 49]. Extracellular S100A4 changes the differentiated state of adjacent cells through damage-associated signaling, which regulates numerous cellular processes crucial to fibrotic progression depending on the location of S100A4 [28]. The initiation of S100A4 expression in epithelial cells that have undergone EMT promotes the generation of extracellular matrix (ECM) components, such as collagen, elastin, and other proteins, providing a basis for the onset of tissue fibrosis [50]. Fibroblast transcription site-1 exists in the promoters of EMT-associated genes, such as Twist, Snail, and β-catenin. It regulates the expression of S100A4 in fibroblast-regulated intestinal and pulmonary tissue fibrosis [51, 52]. Research indicates that S100A4 confers idiopathic pulmonary fibrosis lungs harboring fibrogenic mesenchymal progenitor cells with fibrogenicity [53]. Hypertrophic scars in tissues, followed by fibrosis induced by TNF-α, lead to an increase in MMP, S100A4, and vimentin expression, which can be antagonized by bone morphogenetic protein 2/4 [54].

Furthermore, S100A4 stimulates fibroblasts to secrete fibronectin and collagen, thus forming the structural components of the ECM and stimulating their deposition in tissues, contributing to the formation of a pro-inflammatory niche [55, 56]. The high production of another group of ECM regulators, the MMPs, is induced by both extra- and intracellular S100A4, with the extracellular S100A4-mediated stimulation of MMP being the major path of action [40]. Different MMPs not only promote degradation of the ECM, but also together with other active factors that are stimulated by S100A4 facilitate remodeling of the ECM, inducing a pro-inflammatory microenvironment.

### S100A4 enhances cell migration and chemotaxis

Research has suggested that S100A4 is a potent trigger in pro-inflammatory pathways. High expression of S100A4 enhances the physical motility of macrophages and neutrophils and promotes the recruitment and chemotaxis of these inflammatory cells; this occurs in response to the secretion of inflammatory cytokines [57]. The intra- and extracellular biological functions of S100A4 commonly induce a pathological inflammation-like process in various inflammatory disorders. By interacting with intracellular cytoskeleton–associated target molecules, S100A4 facilitates remodeling of acto-myosin filaments, thus enhancing cell motility and chemotaxis [13]. This contributes to the aggravation of pathological processes via the infiltration of fibroblasts as well as immune and vascular cells into the affected regions, releasing inflammatory factors [58]. Under various pathological stimuli, numerous inflammatory cells upregulate the expression of S100A4 and release it in the form of plasma membrane-derived microvesicles into the extracellular space [59]. The inflammation-associated signal transduction by S100A4 is associated with numerous receptors, such as receptor for advanced glycation end products (RAGE), Toll-like receptor 4, and interleukin (IL)-10 receptor [60–62]. Extracellular S100A4 activates a major pro-inflammatory pathway in the cell, i.e., the MAPK pathway, which triggers the recruitment of cells involved in inflammation and the self-amplifying pro-inflammatory cycle by upregulating several pro-inflammatory cytokines, including IL-1β, IL-6, and TNF-α. Other than cytokines, acute phase reactants, granulocyte colony-stimulating factor, and the well-known inflammation-associated S100 family members, S100A8 and S100A9, are also upregulated, establishing an inflammatory milieu [63]. Additionally, extracellular S100A4 exerts a strong influence on the activation of another major pro-inflammatory transcription factor, NF-κB [61]. Moreover, remodeling of the ECM and expression of aberrant ECM molecules can be stimulated by S100A4, which, in turn, plays a substantial role in supporting the chronic inflammatory response at the affected site [64]. S100A4 also promotes colitis development via S100A4-mediated host inflammatory responses by increasing adhesion and colonization of *Citrobacter rodentium* [29].

S100A4 has been reported to be associated with leukocyte migration, which stimulates cytokine production, particularly, that of granulocyte colony-stimulating factor and eotaxin-2, by T lymphocytes involved in allergic inflammation [65, 66]. The chemo-attracting activity is mediated by extracellular S100A4 via its interactions with specific receptors, such as RAGE [40]. In this manner, S100A4 acts as an important modulator of T cell migration during immune responses, specifically in T-cells [67, 68]. The absence of intracellular S100A4 leads to over-assembly of acto-myosin complexes, resulting in inhibition of chemotactic motility in cultured bone marrow-derived macrophages [57]. Recently, researchers observed that only memory T-cells expressed S100A4. Furthermore, sub-populations of effector memory T-cells showed the highest expression of S100A4. Interestingly, S100A4-deficient memory T-cells also migrated towards chemokines, facilitating autoimmune inflammatory disorders; this is in sharp contrast to the observation that loss of S100A4 expression decreased chemokine-stimulated motility [68]. S100A4 expression is also known to be involved in the pathogenesis of several autoimmune diseases, such as rheumatoid arthritis, systemic sclerosis, and psoriasis. It is markedly upregulated in the joint synovial tissue and also exists in the plasma, while maintaining a bioactive multimeric conformation, in rheumatoid arthritis patients [39]. The levels of the bioactive form of S100A4 are correlated with its disease activity, and thus S100A4 is successfully decreased by blocking therapy in rheumatoid arthritis patients [69].

### S100A4 facilitates neuroprotection

S100A4 plays a pro-survival role in the brain; because of this role, the protein is also considered a neuroprotective factor that protects many types of cells during brain injury through several signaling pathways [21, 60]. The expression of S100A4 in astrocytes not only contributes to the normal process of myelination, but also modulates post-traumatic events in the nervous system. Its expression has been detected in white matter astrocytes at brain trauma sites [70] and was also observed to be markedly upregulated in the hippocampus after excitotoxic injury [60]. In contrast, grey matter astrocytes were observed to be consistently S100A4-negative even after injury [71]. Moreover, S100A4 was found to be clearly overexpressed in astrocytes of the spinal cord adjacent to the injury site [72]. In vitro, S100A4 is secreted by astrocytes to exert its extracellular effects associated with neurite outgrowth and survival of primary hippocampal and cerebellar neurons [60, 73].

S100A4 exerts a neuroprotective pro-survival effect on neurons by rescuing them from brain injury via the Janus kinase/signal transducer and activator of transcription pathway in part by interacting with IL-10R [60]. In a previous study, mimicking S100A4-induced neuroprotection in vivo increased neuronal loss after traumatic brain injury and helped identify two neurotrophic motifs in the S100A4 sequence: H3 and H6 [60]. Thus, S100A4 stimulated the neuritogenesis and survival of cultured neurons. Furthermore, S100A4 protects neurons from an injured central nervous system by neuritogenic and pro-survival effects and likely by promoting glial–axonal interactions [60]. In the peripheral nervous system, the expression of S100A4 increases in the myelinated Schwann cells and unmyelinated Remak bundles at the injury site following a dorsal root or peripheral nerve injury. Its expression also increases in a sub-population of neurons (mainly sensory and autonomic) [74]. Similar to the central nervous system, S100A4 exerts pro-regenerative and pro-survival effects in an injured peripheral nervous system, and mimetic peptides derived from neurotrophic motifs of S100A4 contribute to axonal sprouting and survival [75].

### S100A4 regulates angiogenesis

Recent studies linked S100A4 to several metastatic diseases. One of its metastasis-promoting mechanisms is its function as a potent stimulator of angiogenesis [76]. Angiogenesis is a multi-step process of degradation of the basement membranes and ECM by MMPs, followed by migration of endothelial cells towards the angiogenic stimuli. Next, cell proliferation takes place, followed by formation of a tubular structure that sprouts from pre-existing blood vessels [77]. Research has shown that S100A4 is a pro-angiogenic factor that participates in angiogenesis, endothelial cell migration and invasion via its interaction with annexin 2, formation of plasmin, and stimulation of MMP production [78, 79]. S100A4-mediated angiogenesis also functions synergistically with the vascular endothelial growth factor, which is an important angiogenic factor that promotes neo-vascularization and vascular leakage through an upstream regulator, the brain-derived growth factor [53, 78].

In vivo studies have shown that aging S100A4-transgenic mice have higher chances of acquiring hemangioma compared to non-transgenic mice, and the implantation of S100A4-containing micropellets induce neovascularization in the mouse cornea [22]. In vitro experiments have indicated that S100A4 exogenously applied in cell culture can stimulate the motility of endothelial cells and does not influence the proliferation of these cells, demonstrating that S100A4 acts only in cooperation with other angiogenic factors such as vascular endothelial growth factor and brain-derived growth factor to achieve maximum angiogenesis. Moreover, S100A4 is thought to stimulate endothelial cell motility in a cell-specific manner only because of its inefficacy in promoting the motility of mouse fibroblasts. This indicates that S100A4 identifies a specific receptor on the surface of endothelial cells to help them move [22]. Recently, RAGE located on the surface of human endothelial cells was reported to be utilized by S100A4 to enhance endothelial cell migration and neovascularization. This helps the cells associated with other angiogenic factors and achieve angiogenesis-related responses [53, 80].

## S100A4 is involved in several common non-tumor diseases and the potential clinical application

The promotion of tissue fibrosis by S100A4 has been reported in chronic obstructive pulmonary disease (COPD), pulmonary arterial hypertension (PAH), and cardiac hypertrophy. A recent study by Reimann and colleagues showed that S100A4 expression was increased in the vasculature tissues of COPD patients and in tissues of murine lungs with vascular remodeling. However, there is no clear evidence regarding how S100A4 contributes to vascular remodeling in COPD patients [81]. The complex programs of EMT and endothelial–mesenchymal transition (EndMT) likely contribute to the pathology of COPD because they actively accompany the increase in S100A4 expression in COPD lungs [82]. S100A4 is not only widely reported to be upregulated in fibrosis, but also a key protein regulating the EMT and EndMT processes [83–87]. Increased expression of S100A4 during EndMT in COPD patients may be the mechanism by which primary arterial smooth muscle cells contribute to pulmonary vascular remodeling [88]; this association was made after determining that transitioning endothelial cells stained positively for S100A4.

S100A4 was found to be involved in the pathogenesis of both human and experimental PAH, which is a progressive disease related to pulmonary vasculature construction and remodeling. Here, S100A4 expression was observed to increase in the adventitia and neointima of early occlusive and plexiform lesions in PAH patients [89]. Similarly, in another study, overexpression of S100A4 in mice was reported to increase right ventricular systolic pressure [90], while some mice underwent pulmonary arterial remodeling similar to occlusive and plexogenic lesions that are typically formed in humans [89]. In a recent report, female S100A4-positive mice readily developed PAH, which may be attributable to the increase in S100A4 expression in these female mice [91]. The researchers considered 17β-estradiol/S100A4 RAGE to be responsible for the development of PAH and gender bias in this disease. This conclusion was based on the fact that in conjunction with RAGE activation, 17β-estradiol upregulates S100A4 expression and proliferation of human pulmonary artery smooth muscle cells [91].

S100A4 has also been reported to be upregulated in cardiac hypertrophy [92], which is a complex process involved in numerous cellular events, such as cytoskeletal and/or ECM reorganization, energy metabolism, signal transduction, gene expression, and cardiomyocyte apoptosis [93]. Expression of S100A4 increases in various cardiac cell types, such as cardiomyocytes, cardiac fibroblasts, and immune cells. In cardiac fibroblasts, S100A4 co-localizes with p53 and regulates its target genes, which are associated with cell proliferation and collagen expression. In addition, co-expression of S100A4 and p53 promotes an overload of cardiac pressure that induces cardiac fibrosis; under this condition, S100A4 likely plays a major role in the pro-fibrotic proliferation and maturation of cardiac fibroblasts [94]. In vitro, S100A4 induces cardiomyocyte hypertrophy and increases cell survival, which inhibit apoptosis via MAPK activation and phosphorylation of extracellular signal-regulated kinase 1/2 [41]. In humans, S100A4 is infrequently detected in coronary arteries, while it is markedly expressed in smooth muscle cells of patients suffering from coronary atherosclerosis and coronary restenosis, suggesting that the expression level of S100A4 in smooth muscle cells has significant potential to aid in the risk assessment of coronary atherosclerosis and coronary restenosis [95].

S100A4 is likewise up-regulated in the skin lesions of systemic sclerosis patients. Amelioration of fibrosis symptoms was observed in S100A4-deficient mice from amongst different experimental fibrosis mouse models [56]. Furthermore, S100A4 is released from the upper dermal compartments of psoriatic skin, while S100A4-specific antibodies reduce vascularization, keratinocyte proliferation, and thickness of psoriatic skin [96]. Significant upregulation of S100A4 has been detected in other autoimmune diseases, such as idiopathic inflammatory myopathies [63] and fibrostenosing Crohn’s disease [97]. Moreover, elevated S100A4 expression in human articular chondrocytes during rheumatism and osteoarthritis leads to increased phosphorylation of protein tyrosine kinase-2 and MAPKs; it further activates NF-κB, increasing secreted MMP13 levels [40]. These effects are regulated partly through RAGE, and thus inhibition of RAGE would negatively impact S100A4-dependent signaling pathways [40]. Analysis of bone tissue from osteoarthritis patients using microarrays and quantitative PCR indicated elevated expression of S100A4 as well as that of other wingless-type mouse mammary tumor virus integration site family-related proteins [98].

Elevated S100A4 expression leads to the development and progression of many non-tumor diseases, indicating that targeting of S100A4 expression or activities is a novel strategy for treating non-tumor pathologies. Because of the increase in S100A4 expression in remodeled intrapulmonary arteries, neutralizing antibodies of S100A4 may prevent vascular remodeling of lung fibrosis [37, 81]. The expression of S100A4 is low in the adult normal myocardium, but significantly enhanced in myocardial infarction, and the detection of plasma-S100A4 serves as a novel biomarker for acute myocardial infarction [99]. Cardiac myocyte-specific overexpression of S100A4 after myocardial infarction may protect the infarcted myocardium against myocardial ischemia, while deletion of S100A4 increases cardiac damage [36]. Extracellular S100A4 regulates bone formation in inflammatory bone disease, increasing the risk of fractures and delaying bone healing, and treating primary calvarial osteoblasts with recombinant S100A4 reduces matrix mineralization [100]. Additionally, S100A4 can be a target for the treatment of periodontitis, which inhibits osteogenic differentiation and enhances matrix degradation [35]. The study showing that silencing of the mouse S100A4 gene ameliorates retinal neovascularization in a mouse model indicates that knockdown of S100A4 may be an effective treatment for ocular neovascularization diseases [53]. Furthermore, therapy targeting S100A4 can suppress the progression of rheumatoid arthritis and persistent high S100A4 expression will predict poor treatment outcome of rheumatoid arthritis [101].

## Future perspectives

The roles of S100A4 in cancer progression and metastasis have been demonstrated in numerous studies; this review highlights the participation of S100A4 in the pathophysiologies of several human disorders. The diverse cellular functions of S100A4 and how it exerts its intra- and extracellular influence in non-tumor pathologies remain unclear. As an important cross-linking factor that participates in different signaling pathways, S100A4 participates in mechanisms that are common under different pathological conditions, such as type II EMT in inflammation and fibrosis. S100A4-mediated type II EMT explains several non-tumor biological diseases and the mechanisms by which disease progression is promoted. However, a better understanding of the molecular mechanisms by which S100A4 promotes the progression of these disorders is required. A priority area in studies of S100A4 is to develop novel treatment strategies targeting S100A4 through anti-S100A4 compounds for the therapy of non-tumor diseases.

## Conclusions

In addition to malignant tumors, S100A4 plays an important role in many kinds of pathological process and non-tumor diseases including fibrosis, inflammation, immune response, neuroprotection, and angiogenesis.

## Abbreviations

BDNF: brain derived growth factor
BMP: bone morphogenetic protein
CNS: central nervous system
COPD: chronic obstructive pulmonary disease
ECM: extracellular matrix
EMT: epithelial–mesenchymal transition
EndMT: endothelial–mesenchymal transition
ERK: extracellular signal-regulated kinase
G-CSF: granulocyte colony stimulating factor
JAK: Janus kinase
JNK: jun n-terminal kinase
FSP1: fibroblast-specific protein 1
hPASMCs: human pulmonary artery smooth muscle cells
IL-10R: interleukin-10 receptor
MAPKs: mitogen-activated protein kinases
MET: mesenchymal–epithelial transition
MMP: matrix metalloproteinase
Mts1: metastasin NF-κB: nuclear factor-kappa B
NMIIA: non-muscle myosin
IIA PAH: pulmonary arterial hypertension
PNS: peripheral nervous system
Pyk-2: protein tyrosine kinase-2
RAGE: receptor of advanced glycation end products
RVSP: right ventricular systolic pressure
STAT: signal transducer and activator of transcription
TLR4: toll-like receptor 4
TNF-α: tumor necrosis factor-alpha
VEGF: vascular endothelial growth factor
Wnt: wingless-type mouse mammary tumor virus integration site family
